# Is diversity harmful?—Mixed-brand cardiac implantable electronic devices undergoing magnetic resonance imaging

**DOI:** 10.1007/s00508-021-01924-w

**Published:** 2021-08-17

**Authors:** Christoph Alexander König, Florian Tinhofer, Thomas Puntus, Achim Leo Burger, Nikolaus Neubauer, Herbert Langenberger, Kurt Huber, Michael Nürnberg, David Zweiker

**Affiliations:** 1 3rd Department of Medicine, Cardiology and Intensive Care Medicine, Klinik Ottakring (Wilhelminenhospital), Montleartstraße 37, 1160 Vienna, Austria; 2 Medical University of Vienna, Vienna, Austria; 3 Institute for Diagnostic and Interventional Radiology, Klinik Ottakring (Wilhelminenhospital), Vienna, Austria; 4 Medical School, Sigmund Freud University, Vienna, Austria; 5 Division of Cardiology, Medical University of Graz, Graz, Austria

**Keywords:** Magnetic resonance imaging, Pacemaker, Defibrillator, MRI-conditional, Safety

## Abstract

**Background:**

Many patients with cardiac implantable electronic devices (CIED) undergo magnetic resonance imaging (MRI); however, a relevant proportion have a CIED system that has not been classified as MRI-conditional because of generators and leads from different brands (mixed-brand group). The available data concerning the outcome of these mixed patients undergoing MRI is limited.

**Methods:**

A retrospective single center study, including all patients with CIEDs undergoing MRI between January 2013 until May 2020, was performed. Primary endpoints were defined as death or any adverse event necessitating hospitalization or CIED revision. Secondary endpoints were the occurrence of any sign for beginning device or lead failure or patient discomfort during MRI.

**Results:**

A total of 227 MRI examinations, including 10 thoracic MRIs, were carried out in 158 patients, with 1–9 MRIs per patient. Of the patients 38 underwent 54 procedures in the mixed-brand group and 89 patients underwent 134 MRIs in the MRI-conditional group. Of the patients 31 were excluded since the MRI conditionality could not be determined. No primary endpoints occurred within the mixed-brand group but in 2.2% of the MRI-conditional group (*p* = 1.000), with 2 patients developing new atrial fibrillation during MRI, of whom one additionally had a transient CIED dysfunction. No secondary endpoints were met in the mixed-brand group compared to 3.4% in the MRI-conditional group (*p* = 0.554). No complications occurred in the excluded patients.

**Conclusion:**

The complication rate of CIED patients undergoing MRI was low. Patients with a mixed CIED system showed no signs of increased risk of adverse events compared to patients with MRI-conditional CIED systems.

**Supplementary Information:**

The online version of this article (10.1007/s00508-021-01924-w) contains supplementary material, which is available to authorized users.

## Introduction

Magnetic resonance imaging (MRI) is an important instrument in radiology and increasingly used in daily clinical practice [1]. More than 30 million MRI scans are performed each year in the European Union [2]. Simultaneously, the number of patients with cardiac implantable electronic devices (CIED), such as pacemakers and defibrillators, is increasing quickly as well. Thus, it is estimated that 50–75% of the patients with an CIED need an MRI once in the lifetime of their device [3]. Previously, MRI was contraindicated in the presence of an CIED [4], as generator failure, new-onset arrhythmia and even death were feared [5–7]. Due to advances in technology, manufacturers started with the development of so-called MRI-conditional CIED systems since 2010, minimizing the risks of undergoing MRI with such a CIED system in place, as long as certain specific conditions are fulfilled [8, 9]. Many studies have already demonstrated that the risks of suffering any side effects when undergoing MRI with an MRI-conditional CIED system in place are negligible. [9–13]. Currently, most of the available CIED systems are considered MRI-conditional; however, by 2011 approximately 1.8 million CIED systems were in use that were not MRI-conditional in the USA alone [14]. Although formally unsuitable for undergoing MRI, the risks for suffering any adverse events with an MRI-unconditional CIED system remain very low, as several studies impressively proved [7, 15–17].

A MRI-conditional CIED system refers to both the generator and the attached leads, which are approved only in a certain combination and the absence of abandoned leads [5]; however, a significant proportion of patients have a combination of per se MRI-conditional elements from different manufacturers (mixed-brand group) [18]. Current guidelines endorse performing MRI in these patients as off-label use, if the value of MRI outweighs the small risk of device failure or damage [19] but clinical data are scarce. Therefore, this monocentric analysis was performed to assess possible complications in patients with a mixed-brand CIED system undergoing MRI compared to patients with completely MRI-certified systems.

## Material and methods

This retrospective cohort study included all patients with a CIED who underwent MRI from January 2013 until May 2020 at a major public hospital (Klinik Ottakring, Vienna, Austria). According to the responsible ethics committee, which approved the study (no. 20-137-VK), no informed consent was necessary.

### Clinical workflow at the institution

Patients with a CIED and the need to undergo an MRI were presented to the institution’s department of cardiology. Based on the clinical indications for the MRI and the type of CIED implanted, a decision was made to accept or cancel the MRI procedure based on an individual risk-benefit analysis. The main criteria for acceptance were the absence of abandoned leads and the therapeutic consequence of the MRI examination. If in doubt, the radiologist and the physician responsible for the indications were contacted and the final decision was made following an interdisciplinary discussion. Before MRI, the CIED system was checked and put into MRI mode (if available) and either asynchronous pacing mode (V00 or D00) or the suspension of pacing therapy (000) was programmed. In patients with an implantable cardiac defibrillator (ICD), antitachycardia treatment was deactivated. and patients were continuously accompanied by trained health personnel. During MRI, the electrocardiogram and oxygen saturation were continuously monitored. Immediately after MRI, the CIED was checked and previous settings were restored. The next follow-up was planned after 3–12 months according to the type of CIED and clinical indications.

All MRI examinations were conducted with an MRI scanner with a magnetic field strength of 1.5 T, a maximum gradient field strength of 45 mT/m and a maximum gradient slew rate of 200 T/m/s using solely receiving coils.

Usually, standard MRI protocols were used, following the current guidelines [5], ensuring a maximum whole body SAR (Specific Absorption Rate) of < 2 W/kg and a head SAR of < 3.2 W/kg, as well as minimizing the number of sequences and the scanning duration.

### Patient groups

Patients were stratified into three groups according to the MRI-conditionality of the patient’s CIED system. All patients who had a completely MRI-conditional system according to the manufacturers’ current recommendations were classified into the MRI-conditional group. Patients with MRI-conditional elements but different manufacturers were included into the mixed-brands group. Remaining patients with CIED elements without or with unclear MRI certification were excluded from the primary analysis, but adverse events were still documented (excluded group).

### Data collection

All patients with CIED who received an MRI between 1 January 2013 and 31 May 2020 were included. For analysis of complications after MRI, the patient’s health records within all Viennese public hospitals were reviewed for admissions for a period up to 1 year after the MRI scan.

### Endpoints

As primary endpoint, the occurrence of any complication of a CIED after MRI requiring an intervention (e.g., device failure, lead failure, device dysfunction, or any other periprocedural or postprocedural complication, such as newly developed atrial fibrillation) was compared in MRI-conditional vs. mixed-brand groups.

Secondary endpoints were the occurrence of any sign for beginning device or lead failure or minor clinical conditions. Changes in lead performance were determined on the difference in parameters between pre-MRI and post-MRI as defined by the American Heart Rhythm Society Consensus Statement [5]. An increase in the pacing lead threshold by 1.0 V, a decrease in the P‑wave or R‑wave amplitude by 50%, a pacing lead impedance change by ± 50 Ω or a high-voltage (shock) lead impedance change by ± 5 Ω were considered relevant.

### Statistical analysis

Results were given as mean (standard deviation), median (interquartile range) or proportion, where appropriate. Bivariate analysis of baseline characteristics and outcome between MRI-conditional vs. mixed-brand groups was performed using parametric and non-parametric tests, where appropriate. Fisher’s exact test was used to compare differences of the occurrence of the primary and secondary outcome between both groups. Data were analyzed based on each patient, but the MRI details section of Tables 1 and 2 were calculated based on each MRI examination. All statistical analyses were performed with R 4.0.3 (The R Foundation, Vienna, Austria).

**Table 1:** Baseline characteristics of all included patients, mixed-brand and MRI-conditional groups

Parameter	All included patients	Mixed-brand group	MRI-conditional group	*P* value
**Demographics**				
Number of patients	127	38	89	N/A
Number of MRI procedures	188	54	134	N/A
Age (years) (interquartile range)	73 (67–80)	77 (73–81)	72 (64–79)	0.003^*^
Female gender	34.6%	39.5%	32.6%	0.542
Height (cm)^a^ (± standard deviation)	173 ± 8	173 ± 6	173 ± 9	0.935
Weight (kg)^a^ (interquartile range)	80 (73–84)	78 (71–80)	82 (75–93)	0.126
**CIED details**				
*CIED type*^a^				0.338
Pacemaker	92.1%	97.4%	89.9%	–
Transvenous ICD	6.3%	2.6%	7.9%	–
Subcutaneous ICD	1.6%	0%	2.2%	–
*Indication*^a^				< 0.001
Sick sinus syndrome	30.8%	17.2%	35.9%	–
Atrioventricular block	27.1%	20.7%	29.5%	–
Bradycardia-tachycardia syndrome	23.4%	37.9%	17.9%	–
Other block	4.7%	13.8%	1.3%	–
Heart failure	3.7%	3.4%	3.8%	–
Secondary prophylaxis	3.7%	3.4%	3.8%	–
Other Indication	6.5%	3.4%	7.7%	–
*Generator brand*^a^				< 0.001
Biotronik^b^	32.3%	5.3%	43.8%	–
Boston Scientific^c^	12.6%	10.5%	13.5%	–
Medtronic^d^	24.4%	36.8%	19.1%	–
Sorin/LivaNova^e^	17.3%	42.1%	6.7%	–
St. Jude Medical/Abbott^f^	13.4%	5.3%	16.9%	–
Months since prior box change before MRI	26.8 (12.1–48.1)	33.0 (11.4–48.9)	25.4 (12.3–47.9)	0.953
Prior box change < 3 months	2.4%	2.6%	2.2%	1.000
*Lead brand*^a^				0.002
Biotronik^b^	54.3%	78.9%	43.8%	–
Boston Scientific^c^	10.2%	2.6%	13.5%	–
Medtronic^d^	18.1%	15.8%	19.1%	–
Sorin/LivaNova^e^	4.7%	0.0%	6.7%	–
St. Jude Medical/Abbott^f^	12.6%	2.6%	16.9%	–
Months since last lead implantation before MRI	36.6 (15.1–66.1)	39.2 (18.0–80.4)	34.3 (14.2–61.6)	0.662
Last lead implantation < 3 months	0.8%	2.6%	0.0%	0.299
**MRI details**				
*Location of MRI examination*				0.322
Head or extremities	40.4%	48.1%	37.3%	–
Extrathoracic torso	55.3%	50.0%	57.5%	–
Thorax	4.3%	1.9%	5.2%	–
*Pacing mode during MRI*^a^				0.681
000	7.4%	10.0%	6.8%	–
A00/V00	15.7%	10.0%	17.0%	–
D00	76.9%	80.0%	76.1%	–
Estimated ERI before MRI (years)	7.4 (6.0–9.3)	7.0 (5.0–10.3)	7.5 (6.0–9.1)	0.831

*CIED* cardiac implantable electronic device, *ICD* implantable cardioverter/defibrillator, *MRI* magnetic resonance imaging, *ERI* elective replacement indicator

^*^ *p* < 0.05

^a^incomplete data for some patients

^b^(Berlin, Germany)

^c^(Marborough, MA, USA)

^d^(Dublin, Ireland)

^e^(London, UK)

^f^(St. Paul, MN, USA)

**Table 2:** Outcome of included patients, mixed-brands and MRI-conditional groups

Parameter	All included patients (%)	Mixed-brands group (%)	MRI-conditional group (%)	*P* value
**Shot-term outcome**				
*Primary endpoints*	1.6	0.0	2.2	1.000
*Secondary endpoints*	2.4	0.0	3.4	0.554
Patient discomfort	1.6	0.0	2.2	1.000
Increase of ventricular threshold	0.8	0.0	1.1	1.000
**Long-term follow-up**				
Decrease of amplitude > 50%	4.7	7.9	3.4	0.363
Increase of pacing threshold > 1 V	3.1	0.0	4.5	0.316
1‑year rehospitalization	37.8	36.8	38.2	1.000

*CIED* cardiac implantable electronic device, *ICD* implantable cardioverter/defibrillator, *MRI* magnetic resonance imaging

^*^ *p* < 0.05

## Results

**Fig. 1:**
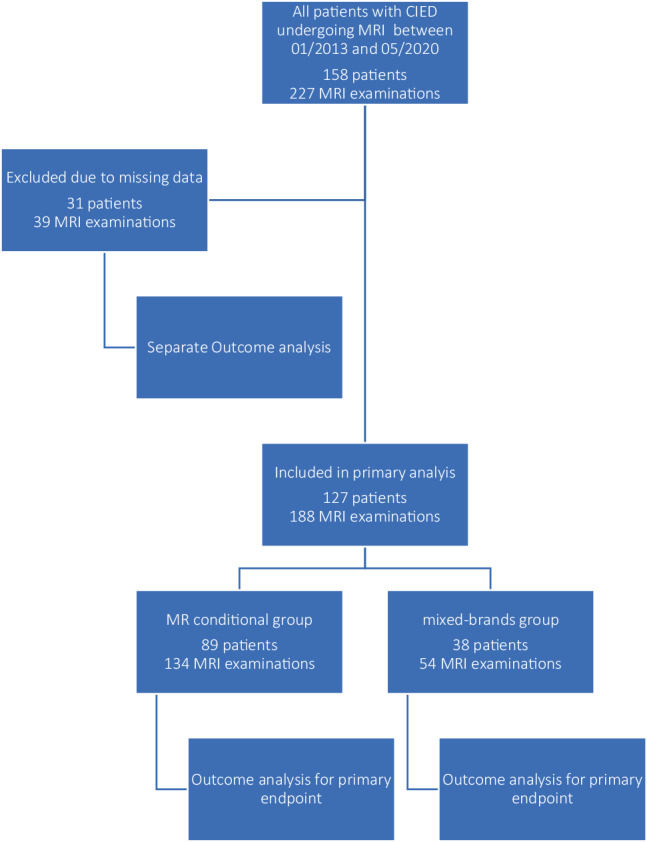
Study flowchart. *CIED* cardiac implantable electric device, *MRI* magnetic resonance imaging

In the study period, a total of 227 MRI examinations were performed in 158 different CIED patients, with a range of 1–9 MRI procedures per patient. The total yearly number of MRI examinations rose from 15 in 2013 to 64 in 2018. Of all procedures, 134 MRI procedures in 89 patients were stratified into the MRI-conditional group, while 54 MRI procedures in 38 patients were included in the mixed-brand group. The MRI-conditionality could not be determined in 31 patients undergoing 39 MRI procedures and were therefore excluded from the primary analysis. In the MRI-conditional group, 7 patients (5.2%) underwent a thoracic MRI, including 5 specifically cardiac MRIs. In the mixed-brand group, there was one patient (1.9%) who received a cardiac MRI. There were no MRIs found that were performed on patients with abandoned leads present. A study flowchart is presented in Fig. 1.

### Demographics

The overall mean age was 73 years, with patients in the mixed-brand group being significantly older than patients of the conditional group (77 years vs. 72 years, *p* = 0.003). Mean height was 173 cm, median weight was 80 kg and 34.6% of patients were female, with no significant differences between groups (Table 1). Baseline characteristics of excluded patients are available in Supplemental Table 1.

### CIED details

Within all the included patients, 92.1% of the CIEDs were pacemakers and 7.9% were ICDs. The most common indications for CIED implantation within the included patients were sick sinus syndrome (30.8%), followed by atrioventricular block (27.1%) and bradycardia-tachycardia syndrome (23.4%) (Table 1). In total, within all the included patients, the most common pacemaker brand, concerning both generators (29.9%) and leads (54.7%), was Biotronik (Berlin, Germany). Notable are the significant differences between the different groups, with 42.5% of the pacemaker generators from Biotronik in the MRI-conditional group, compared to only 2.7% pacemaker generators from Biotronik in the mixed-brands group, where most generators (43.2%) were from Sorin/Liva Nova (London, United Kingdom) instead. The lead brands too showed significant differences between the different groups, with 42.5% pacemaker leads from Biotronik in the MRI-conditional group, compared to 81.1% pacemaker leads, also from Biotronik, in the mixed-brands group. Additional details concerning generator and lead distribution are found in Supplemental Table 2.

For ICDs, solely generators from Biotronik (60.0%) and Boston Scientific (Natick, MA, USA) (40.0%) were used, with leads mostly from Biotronik (50.0%), followed by Boston Scientific (40.0%). Here too, additional CIED details, stratified by pacemakers and ICDs, as well as groups, are given in Supplemental Table 2.

The time between the last generator or lead implantation and the MRI was on average 32.5 months and 45.3 months, with 1.6% and 0.5%, respectively, having received the last procedure within the last 6 weeks. The mean time of the elective replacement indicator (ERI) was 7.5 years.

### Short-term outcome

Primary endpoints were met in two patients (1.6%), with no endpoints in the mixed-brand group (0.0%) and two in the MRI-conditional group (2.2%, *p* = 1.000, Table 2). One patient with a dual-chamber CRT‑D, receiving a cranial MRI, developed de novo atrial fibrillation with fast ventricular conduction during the MRI examination. During the postprocedural CIED examination, the impedance of the right ventricular defibrillation electrode was not measurable and a marked drop in battery capacity above the elective replacement indicated (ERI) threshold was noted. The patient was hospitalized for one night until spontaneous conversion into sinus rhythm. The treating clinicians suspected the MRI pacing mode (D00 instead of 000 despite spontaneous sinus rhythm) to have caused the arrhythmia; however, the CIED examination before discharge showed normal measurements regarding the battery and electrodes. Since the atrial fibrillation was considered to be triggered by CIED dysfunction, no anticoagulation treatment was established. At follow-up no further atrial arrhythmias were documented.

In another case, the device check following an unremarkable knee MRI examination revealed de novo atrial fibrillation with activation of mode switch. Anticoagulation treatment had already been established previously due to an indication other than atrial fibrillation and the follow-up after 1 year showed no further atrial arrhythmias.

In the short-term outcome, secondary endpoints occurred in three patients during three MRI examinations (2.4%), also solely in the MRI-conditional group (*p* = 0.554). One patient with a lumbar MRI developed stenocardia when switching the CIED into the MRI mode. In another patient, also during a lumbar MRI, the MRI had to be aborted because the patient felt heat in the generator area during the examination. Both patients had an unremarkable follow-up. No other patients showed any symptoms when undergoing MRI. In a third patient, an increase of the ventricular threshold was noted during a routine control 6 months after the MRI examination of the shoulder.

### Long-term outcome and changes in lead performance

The long-term follow-up further revealed four additional cases of threshold increase (3.1%) during the follow-up, several months after the MRI examination. All events occurred in patients with MRI-conditional CIEDs (4.5%) compared to none in the mixed-brands group (0%, *p* = 0.316) Additionally, 6 more cases (4.7%) of sensing amplitude decrease by > 50% were identified, 3 in the MRI-conditional and 3 in the mixed-brands group (3.4% in MRI-conditional group vs. 7.9% in mixed-brands group, *p* = 0.363). More details concerning the lead performance are given in Supplemental Table 3.

In a period of 1 year after MRI, 38% of patients were hospitalized, without significant differences between groups (38.2% vs. 36.8%, *p* = 1.000).

### Events in excluded patients

In the patient group where the MRI-conditionality could not be determined, neither primary nor secondary endpoints were met, nor were any changes in lead performance noticed.

## Discussion

In this retrospective monocentric study, the safety of MRI-unconditional CIED systems due to mixed-brand components was compared with MRI-conditional CIED systems in daily clinical practice. The study included consecutive patients with a CIED undergoing MRI at our institution and found no sign of increased adverse events in the mixed-brand group.

### MRI and CIEDs

The possible interactions between the MRI unit and implantable electronic devices are diverse and must be considered carefully by treating physicians, as the possible risks a CIED patient is facing when undergoing MRI are many [20–22], which is why a short summary is provided here.

There are three basic physical forces that can cause electromagnetic interferences (EMI) of the CIED, namely the static magnetic field, time varying gradient magnetic fields and radiofrequency energies. The static magnetic field affects the ferromagnetic parts of the CIED and may lead to distorting or loosening of the implanted parts [23] or may affect the so-called reed switch [24] that can still be found in older CIEDs. Gradient magnetic fields on the other hand may induce currents in conductive wires and thus may lead to unwanted myocardial stimulation and induce arrhythmias [25]. The radiofrequency energy, however, may lead to excessive heating of the local tissue [26], possibly even leading to tissue damage, especially when causing a so-called antenna effect [27]. Other risks to keep in mind are battery depletion [28] or power-on reset (POR) [29] as well as imaging artefacts that may occur when performing MRI in regions with a CIED in situ, such as thoracic or cardiac MRI [30].

While the benefits of unitary CIED systems from one manufacturer are obvious, the combination of different brands often cannot be avoided. Reasons may range from unavailability of elements from specific brands up to the preference of the implanting physician. As current CIEDs have to conform with specific requirements [31] ensuring compatibility, patients with mixed-brand systems normally are not facing problems; however, in the era of MRI-conditional CIED devices, new challenges arise for both the patients and the treating physicians. While the decision to perform the MRI may be backed up by guidelines [5, 19], the use of CIED devices as off-label and the legal consequences should be kept in mind. This study shows that a high proportion (30.2%) of patients are now affected by this problem.

There are only a handful of comparative studies, focusing on the different outcomes between MRI conditional vs. MRI unconditional CIEDs. Notably, there is Shah et al. from 2017, including 105 patients but without a follow-up investigation [32], then there is Han et al. in 2019 with a total of 35 patients and a follow-up of 1 month after MRI [33] and last there is Seewöster et al. from 2019, comparing a total of 200 consecutive patients, with a follow-up interrogation 6 months after the MRI examination [34]. None of these studies showed any significant differences between groups nor any significant adverse effects in general when undergoing MRI [32–34].

Those studies did not take into consideration that CIED systems using elements from different manufacturers (although the separate parts themselves are per se MRI-conditional) are formally not considered as MR-conditional as a whole [5] but should rather be considered as MRI-unconditional and thus the performing of MRI in these patients remains an off-label use.

To the authors knowledge, this is the first study to investigate the effect of mixed brand CIED systems undergoing MRI, incorporating detailed information on device and lead measurements; however, although the presence of mixed-brand systems represents novelty, the results are not surprising as the single components themselves are all MRI-conditional and there are several studies that already proved the relative safety of undergoing magnetic resonance imaging even with an MRI-unconditional CIED [7, 15–17]. Additionally, only standard MRI protocols were used, which is a further reason no adverse events were to be expected, as perhaps would have been the case when performing MRIs with a higher energy deposition or closer to the generator.

Fortunately, the results suggest that undergoing MRI was safe even in these mixed-brands group, with two primary endpoints met solely in the MRI-conditional group. Both times the patients developed de novo atrial fibrillation. Both patients had a CIED system implanted that was MRI-conditional with generators and leads from the same manufacturer. The rate of secondary endpoints was also low. No device or lead replacement was necessary after MRI. These results, demonstrating the relative safety of undergoing MRI with a CIED, coincide with other studies already conducted, both for MRI-conditional [9–13] and unconditional CIEDs [7, 15–17].

In general, there were no significant differences noted between pre-MRI and post-MRI parameters or between the MR-conditional and the mixed-brands group, concerning capture threshold, sensing or impedance. Most changes were in a clinically acceptable range and coincided with other large studies in terms of the type of observed events [7, 9–13, 15–17].

The study included a (albeit small) number of thoracic and cardiac MRIs. No endpoints were met in these patients, neither in the MRI-conditional nor the mixed-brand group. Even though the significance of these results is small, due to the small number of cardiac MRIs observed, the results agree with other studies with bigger sample sizes that also showed that cardiac magnetic resonance is possible on patients with CIEDs [34–36]. Whether any imaging artifacts occurred with these thoracic MRIs or not, was not assessed by this study.

In addition, this study included many patients with repeated MRI examinations and the effect of multiple MRIs on the CIED systems could be examined. Undergoing multiple MRI examinations did not seem to have any influence on the outcome, since the few observed endpoints occurred both with patients who received multiple MRIs as well as with patients who received only one single MRI examination. This is corresponding with previous studies that also included multiple MRI examinations [13, 16, 37] and showed that patients who underwent multiple MRIs (the maximum number in a single patient was 9 MRIs) did not suffer any adverse effects; however, the study cannot add much evidence on the effect of multiple MRI examinations in patients with mixed-brand ICDs, as there was only one patient with a mixed-brand ICD who underwent two MRI examinations without adverse effects.

## Limitations

The main limitation is the retrospective nature of this study. Due to missing data, only 134 MRIs could be allocated to the MRI-conditional group and 54 MRIs into the mixed-brands group. 39 MRIs that had to be excluded from this study, since their MRI-conditionality could not be determined due to missing data. Fortunately, in uncategorized patients none of the discussed endpoints occurred.

Another limitation is certainly the imbalance between pacemakers and ICDs. With only one single ICD patient in the mixed-brand group, these results may not be transferred to other populations with a higher proportion of ICD patients.

Additionally, we faced difficulties collecting all the individual lead parameters during the follow-ups, due to ofttimes missing data; however, in the data available, no significant abnormalities were found.

## Conclusion

Our analysis did not find any evidence of increased risk of adverse events in patients with mixed-brand CIED systems undergoing MRI compared to patients with MRI-conditional CIED systems. Overall, it can be concluded that when a CIED patient is properly examined according to the established guidelines, undergoing MRI is relatively safe, regardless of whether the patient undergoes MRI with an MRI-conditional or a formally unconditional mixed CIED system, using MRI-conditional components from different manufacturers.

After all, this study shows that diversity appears not to be harmful at all.

## Supplementary Information

The supplemental material provides further details on the number of the performed MRI-examinations each year, as well as baseline characteristics of the excluded patients. Furthermore, the CIED details of all patients are given in more detail. Also, the changes in lead performance, compared by individual groups and at different times of the follow-up visit, are included.
