# Supplementary material for: Is diversity harmful?—Mixed-brand cardiac implantable electronic devices undergoing magnetic resonance imaging
Source: Wien Klin Wochenschr. 2021 Aug 17;134(7-8):286–93. doi: 10.1007/s00508-021-01924-w (PMC9023390; doi:10.1007/s00508-021-01924-w)
Supplement: Supplementary file 1 — The supplemental material provides further details on the number of the performed MRI-examinations each year, as well as baseline characteristics of the excluded patients. Furthermore, the CIED details of all patients are given in more detail. Also, the changes in lead performance, compared by individual groups and at different times of the follow-up visit, are included. [file 508_2021_1924_MOESM1_ESM.pdf]

Supplemental Figure 1. MRI-Examinations stratified by years, “MRI-conditional”, “mixed-brands” and “MRIs with unknown CIEDs” groups.

Year	MRIs with MR-conditional CIEDs	MRIs with mixed-brands CIEDs	MRIs with unknown CIEDs	total
2013	10	5	0	15
2014	7	4	1	12
2015	8	6	0	14
2016	25	9	2	36
2017	26	14	9	49
2018	39	7	19	64
*2019**	18	8	6	32
*2020**	2	1	2	5
**total**	131	54	42	227

CIED: cardiac implantable electronic device, MRI: magnetic resonance imaging
*The years 2019 and 2020 were not fully examined by our study

Supplemental Table1. Baseline characteristics of excluded patients.

Parameter	*Excluded patients*
Demographics	
Number of patients	31
Number of MRI procedures	39
Age	71 (63-80)
Female gender	25.8%
Height (cm)†	169±14
Weight (kg)†	83 (81-98)
CIED details	
CIED type†	
Pacemaker	67.7%
ICD	12.9%
Unknown	19.4%
Indication†	
Atrioventricular block	16.7%
Brady tachy syndrome	16.7%
Sick sinus syndrome	0.0%
Other block	0.0%
Heart failure	33.3%
Secondary prophylaxis	16.7%
Other Indications	16.7%
***Generator brand*†**	
Biotronik	31.6%
Boston Scientific	5.3%
Medtronic	42.1%
Sorin/LivaNova	0.0%
St. Jude Medical/Abbott	21.1%
Months since prior box change before MRI	19.9 (9.8-43.7)
Prior box change < 1.5 months	0.0%
MRI details	
Location of MRI examination†	
Head or extremities	48.7%
Extrathoracic torso	46.2%
Thorax	5.1%
Pacing mode during MRI†	
000	4.5%
A00/V00	18.2%
D00	77.3%
Estimated ERI before MRI (years)	8.1 (5.8-11.0)

CIED: cardiac implantable electronic device, ICD: implantable cardioverter/defibrillator, MRI: magnetic resonance imaging, ERI: elective replacement indicator.

* $p<0.05$; † data not complete in all patients.

Supplemental Table 2. CIED details of all included patients, mixed-brand and MRI-conditional groups - divided into pacemakers and ICDs.

Parameter	*All included patients*	*mixed-brand group*	*MRI-conditional group*	*P value*
**CIED details**				
CIED type†				0.338
Pacemaker	92.1%	97.4%	89.9%	
Transvenous ICD	6.3%	2.6%	7.9%	
Subcutaneous ICD	1.6%	0%	2.2%	
Pacemaker details				
***Generator brand*†**				<0.001*
Biotronik	29.9%	2.7%	42.5%	
Boston Scientific	10.3%	10.8%	10.0%	
Medtronic	26.5%	37.8%	21.3%	
Sorin/LivaNova	18.8%	43.2%	7.5%	
St. Jude Medical/Abbott	14.5%	5.4%	18.8%	
***Lead brand*†**				0.002*
Biotronik	54.7%	81.1%	42.5%	
Boston Scientific	7.6%	2.7%	10.0%	
Medtronic	18.8%	13.5%	21.3%	
Sorin/LivaNova	5.1%	0.0%	7.5%	
St. Jude Medical/Abbott	13.7%	2.7%	18.8%	
ICD details				
***Generator brand*†**				1.000
Biotronik	60.0%	100.0%	55.6%	
Boston Scientific	40.0%	0.0%	44.4%	
***Lead brand*†**				0.100
Biotronik	50.0%	0.0%	55.6%	
Boston Scientific	40.0%	0.0%	44.4%	
Medtronic	10.0%	100.0%	0.0%	

CIED: cardiac implantable electronic device. ICD: implantable cardioverter/defibrillator. MRI: magnetic resonance imaging.
* p<0,05; † data not complete in all patients.

Supplemental Table 3. Changes in lead performance compared by groups at different follow-ups

Groups / *Lead Parameters*	Total		All-included patients		MRI-Conditional group		Mixed-Brands group		Excluded patients	
	mean change	*standard deviation*	*mean change*	*standard deviation*	*mean change*	*standard deviation*	*mean change*	*standard deviation*	*mean change*	*standard deviation*
	**Difference Pre-MRI/Post-MRI**									
RA threshold (V)	-0.0	0.2	+0.0	0.2	-0.0	0.2	+0.1	0.2	-0.2	0.2
RA sensing (%)	+2.8%	23.5	+2.6%	24.9	+2.1%	21.8	+4.9%	36.4	+4.5%	6.1
RA impedance (Ω)	-9.4	56.9	-7.7	52.0	-6.6	52.5	-13.5	50.6	-24,9	93.7
RV threshold (V)	+0.0	0.2	+0.0	0.2	+0.0	0.3	-0.0	0.1	-0.0	0.1
RV sensing (%)	+7.2%	28.6	+7.2%	28.5	+7.2%	22.6	+7.2%	43.1	+7.2%	30.4
RV impedance (Ω)	+6.5	49.1	+7.4	51.4	+2.5	53.1	+26.3	40.1	-1.6	18.3
	**Difference Pre-MRI/1.Follow-up**									
RA threshold (V)	+0.0	0.2	+0.0	0.3	+0.0	0.2	-0.0	0.4	+0.0	0.1
RA sensing (%)	+8.4%	60.9	+9.2%	65.5	+13.8%	71.6	-9.8%	20.9	+3.3%	13.7
RA impedance (Ω)	+4.0	77.2	+3.4	66.9	+3.0	68.6	+5.4	61.4	+7.6	130.9
RV threshold (V)	+0.0	0.3	+0.0	0.3	+0.0	0.3	+0.0	0.3	-0.0	0.1
RV sensing (%)	+7.0%	31.6	+6.9%	32.8	+8.0%	33.3	+3.5%	31.7	+7.8%	24.9
RV impedance (Ω)	+4.2	70.0	+3.8	74.9	-3.2	72.5	+28.2	80.2	+6.9	29.4
	**Difference Pre-MRI/2.Follow-up**									
RA threshold (V)	+0.1	0.3	+0.1	0.3	+0.1	0.3	+0.1	0.3	+0.0	0.2
RA sensing (%)	-3.5%	27.4	-4.1%	27.5	-3.2%	27.6	-7.6%	28.2	+3.8%	27.7
RA impedance (Ω)	+0.4	64.9	-0,5	65.7	+0.0	62.7	-2.1	77.8	+13.8	57.4
RV threshold (V)	+0.0	0.2	+0.1	0.3	+0.0	0.3	+0.1	0.3	+0.0	0.1
RV sensing (%)	-2.0%	29.9	-1.8%	31.1	-0.3%	32.2	-6.1%	28.5	-3.8%	19.4
RV impedance (Ω)	+2.8	78.0	+3.2	80.9	-7.5	77.2	+32.9	85.9	-0.3	54.2

CIED: cardiac implantable electronic device, MRI: magnetic resonance imaging, RA: right atrial, RV: right ventricular
